# The Effect of Initial Oxygen Exposure on Diaphragm Activity in Preterm Infants at Birth

**DOI:** 10.3389/fped.2021.640491

**Published:** 2021-02-09

**Authors:** Ruud W. van Leuteren, Anouk W. J. Scholten, Janneke Dekker, Tessa Martherus, Frans H. de Jongh, Anton H. van Kaam, Arjan B. te Pas, Jeroen Hutten

**Affiliations:** ^1^Department of Neonatology, Emma Children's Hospital, Amsterdam UMC, University of Amsterdam, Amsterdam, Netherlands; ^2^Amsterdam Reproduction and Development Research Institute, Amsterdam, Netherlands; ^3^Department of Neonatology, Leiden University Medical Centre, Leiden, Netherlands; ^4^Faculty of Science and Technology, University of Twente, Enschede, Netherlands; ^5^Department of Neonatology, Emma Children's Hospital, Amsterdam UMC, Vrije Universiteit, Amsterdam, Netherlands

**Keywords:** diaphragm activity, breathing effort, oxygen, preterm infant, delivery room

## Abstract

**Background:** The initial FiO_2_ that should be used for the stabilization of preterm infants in the delivery room (DR) is still a matter of debate as both hypoxia and hyperoxia should be prevented. A recent randomized controlled trial showed that preterm infants [gestational age (GA) < 30 weeks] stabilized with an initial high FiO_2_ (1.0) had a significantly higher breathing effort than infants stabilized with a low FiO_2_ (0.3). As the diaphragm is the main respiratory muscle in these infants, we aimed to describe the effects of the initial FiO_2_ on diaphragm activity.

**Methods:** In a subgroup of infants from the original bi-center randomized controlled trial diaphragm activity was measured with transcutaneous electromyography of the diaphragm (dEMG), using three skin electrodes that were placed directly after birth. Diaphragm activity was compared in the first 5 min after birth. From the dEMG respiratory waveform several outcome measures were determined for comparison of the groups: average peak- and tonic inspiratory activity (dEMG_peak_ and dEMG_ton_, respectively), inspiratory amplitude (dEMG_amp_), area under the curve (dEMG_AUC_) and the respiratory rate (RR).

**Results:** Thirty-one infants were included in this subgroup, of which 29 could be analyzed [*n* = 15 (median GA 28.4 weeks) and *n* = 14 (median GA 27.9 weeks) for the 100 and 30% oxygen group, respectively]. Tonic diaphragm activity was significantly higher in the high FiO_2_-group (4.3 ± 2.1 μV vs. 2.9 ± 1.1 μV; *p* = 0.047). The other dEMG-parameters (dEMG_peak_, dEMG_amp_, dEMG_AUC_) showed consistently higher values in the high FiO_2_ group, but did not reach statistical significance. Average RR showed similar values in both groups (34 ± 9 vs. 32 ± 10 breaths/min for the high and low oxygen group, respectively).

**Conclusion:** Preterm infants stabilized with an initial high FiO_2_ showed significantly more tonic diaphragm activity and an overall trend toward a higher level of diaphragm activity than those stabilized with an initial low FiO_2_. These results confirm that a high initial FiO_2_ after birth stimulates breathing effort, which can be objectified with dEMG.

## Introduction

At birth, lung liquid clearance and aeration are required to ensure adequate gas exchange after the cord is clamped. Spontaneous breathing is one of the key factors in this process (1). In contrast to term infants, up to 70% of preterm infants experience difficulty in making this pulmonary transition, with absent or insufficient respiratory drive as an important cause (2, 3). For this reason, preterm infants often require continuous positive airway pressure (CPAP) or intermittent positive pressure ventilation (IPPV) in combination with supplemental oxygen in the delivery room (DR) to successfully make the transition from intra- to extra-uterine life.

In recent years, various studies have investigated individual interventions to stimulate spontaneous breathing in preterm infants at birth. It has been shown that optimization of the CPAP pressure-level, use of repetitive tactile stimulation, or administering caffein shortly after birth stimulates spontaneous breathing, thereby contributing to a successful transition (4–6). While oxygenation is a major determinant of respiratory drive, the optimal initial fraction of oxygen (FiO_2_) that should be used in the DR is still a matter of debate. Restricted use of oxygen has been advocated in recent guidelines, in an attempt to reduce the risk of injurious hyperoxemia (7). However, using a lower initial FiO_2_ at birth may also lead to hypoxemia which can inhibit breathing effort and thus pulmonary transition (8). This was recently confirmed by a randomized trial (the IMPROvE trial) assessing the effect of an initial high vs. a low FiO_2_ on breathing effort in preterm infants (9). The results showed that infants in the high FiO_2_ (1.0) group had larger tidal volumes, resulting in a significantly higher average minute volume (MV) during the first 5 min after birth, compared to a low FiO_2_ (0.3). The differences between the two FiO_2_ groups confirm that oxygen might be an important mediator of breathing effort after birth.

It is known that respiratory control consists of the respiratory rhythm regulator in the brain stem, the sensory input of central and peripheral oxygen chemoreceptors providing feedback to central regulation, and the muscular effector. Extensive evidence exists of the influence of oxygen and neurotransmitters (e.g., adenosine) on the signaling pathways between sensory input of the chemoreceptors and the respiratory rhythm regulator in the brainstem (8, 10). On the other hand, the effects of initial oxygen exposure on the muscular effector of the system, the diaphragm, are less clear. As the main effector of the system, the improved breathing effort when administering higher concentrations of oxygen is likely to be mediated through an increased diaphragmatic activity. To investigate this mechanism, we measured the electrical activity of the diaphragm via transcutaneous electromyography (dEMG) in a subgroup of the infants included in the IMPROvE trial. A recent study showed that transcutaneous dEMG monitoring is feasible in preterm infants stabilized in the DR (11).

The aim of this study was to determine the effects of an initial high vs. low oxygen level on the activity of the diaphragm in preterm infants and we hypothesized that a higher oxygen level would increase the electrical activity compared to a lower oxygen concentration.

## Methods

This study was part of the IMPROvE trial, a randomized controlled trial conducted in the Leiden University Medical Center (LUMC) and Amsterdam University Medical Center (Amsterdam UMC), both located in the Netherlands (9).

### Study Population and Data Acquisition

Preterm infants born after a gestational age between 24^0/7^ and 29^6/7^ weeks were included in the IMPROvE trial. Exclusion criteria were congenital abnormalities or conditions that could directly affect breathing effort. All infants were randomly allocated to an initial high (1.0) or low (0.3) FiO_2_. During the stabilization in the DR (first 15 min after birth), the oxygen level was titrated according to the Dawson oxygen saturation ranges (12). Respiratory support was provided with a facemask connected to a T-piece resuscitator, following the protocol of the individual departments [based on international guidelines and the Dawson saturation curves (9, 13)]. A respiratory function monitor (RFM) (NewLifeBox, Advanced Life Diagnostics, Weener, Germany) was used to record respiratory data and vital signs in real time within a Polybench software application (Applied Biosignals, Weener Germany).

In 31 of the 50 included infants, diaphragm activity was measured with dEMG. Three skin electrodes (H59P, Covidien, Ireland) were placed on the infant's chest, two bilateral in the midclavicular line at the costal margin and a reference electrode on the sternum. The electrodes were connected to the Dipha-16 signal amplifier (Demcon, Macawi Medical Systems, Enschede, The Netherlands) which measured the raw dEMG-signal at 500 Hz and sent it wirelessly to the RFM. Electrodes were placed as soon as possible after birth and measured diaphragm activity for the first 15 min after birth or until transfer to the ward. A camera placed at the far end of the bed recorded the procedures, in order to explain signal artifacts afterwards.

### Data Analysis

The amplified dEMG signal was post-processed to remove background noise by high-pass and low-pass filtering and cardiac interference was removed with the gating technique (14). The moving average of the resulting signal was calculated and the acquired respiratory waveform was used to describe diaphragmatic activity in the two groups. The entire recording was analyzed, as it offered the opportunity to assess diaphragm activity over time (11). Offline analysis was done in MATLAB (version 2018a, Mathworks, Natick, USA) in a custom-made graphical user interface to facilitate visual inspection of the data.

First, review of the video footage, visual inspection of the signals and a threshold value to identify signal spikes were used to remove major signal artifacts due to e.g., disconnection of the electrode or clinical handling. Subsequently, the cleaned signal was used to derive the following parameters breath-by-breath from the dEMG respiratory waveform: peak (end-inspiratory, dEMG_peak_) and tonic (end-expiratory, dEMG_ton_) activity, inspiratory amplitude (dEMG_amp_) defined as dEMG_peak_ minus previous dEMG_ton_, inspiratory area under the curve (dEMG_AUC_), inspiratory time (T_i_), expiratory time (T_e_) and respiratory rate (RR). Each parameter was averaged per minute, for each infant. If data of a specific minute was not available, due to artifact removal, this minute was discarded.

### Statistical Analysis

All parametric data were presented as mean ± standard deviation and non-parametric data as median (interquartile range). In agreement with the original IMPROvE trial differences in average dEMG-parameters in the first 5 min after birth between the study groups were tested with a Student's *t*-test or with Mann-Whitney *U*-test, according to the distribution of the data. The choice of this time frame was also supported by the finding in the original trial that after 5 min of FiO_2_ titration the contrast in averaged received FiO_2_ between the groups was reduced (9). In all cases a *p* < 0.05 was considered significant. SPSS (version 26, IBM, Chicago, USA) was used for statistical analysis.

## Results

In 31 infants dEMG was measured, of which measurements of two infants were excluded from the analysis due to an error in data storage. This resulted in 29 infants, who were equally distributed between the high (*n* = 15) and low (*n* = 14) oxygen group (Table 1). There were no significant differences between the two groups with respect to gestational age, weight, gender, mode of delivery, use of antenatal steroids, maternal medication or pregnancy complications. IPPV was used more often in the first 5 min in the 30% oxygen group, but this difference did not reach statistical significance. However, infants in the 100% oxygen group showed a significantly higher Apgar score at 1 and 5 min.

**Table 1:** Subject characteristics of the dEMG-subgroup.

	**100% O_**2**_*n* = 15**	**30% O_**2**_*n* = 14**
Gestational age (weeks)	28.4 (26.1–29.0)	27.9 (26.3−28.6)
Birth weight (g)	1,060 ± 314	991 ± 253
Male gender, *n* (%)	7 (53.3)	6 (42.9)
Cesarean section, *n* (%)	11 (73.3)	7 (50.0)
Full course antenatal corticosteroids, *n* (%)	7 (46.7)	8 (57.1)
Maternal medication influencing infant respiration, *n* (%)	1 (6.7)	0 (0)
Complications during pregnancy, *n* (%)	6 (40.0)	5 (35.7)
PPROM	1 (6.7)	1 (7.1)
PIH	3 (20.0)	1 (7.1)
Intra-uterine infection	0 (0)	1 (7.1)
IUGR	0 (0)	2 (14.3)
Multiple	2 (13.3)	0 (0)
Apgar 1 min	6 (6–8)	5 (2–6)^*^
Apgar 5 min	8 (8–9)	8 (6–9)^*^
IPPV (% of first 5 min)	10.9 (0–30.4)	26.8 (3.6–49.8)

All continuous values are expressed as mean ± standard deviation or median (interquartile range). Categorical variables expressed as n (%). PPROM, preterm pre-labor rupture of membranes; PIH, pregnancy-induced hypertension; IUGR, intra-uterine growth restriction; IPPV, intermittent positive pressure ventilation.

* *p < 0.05*.

### Oxygen and Diaphragm Activity

**Figure 1:**
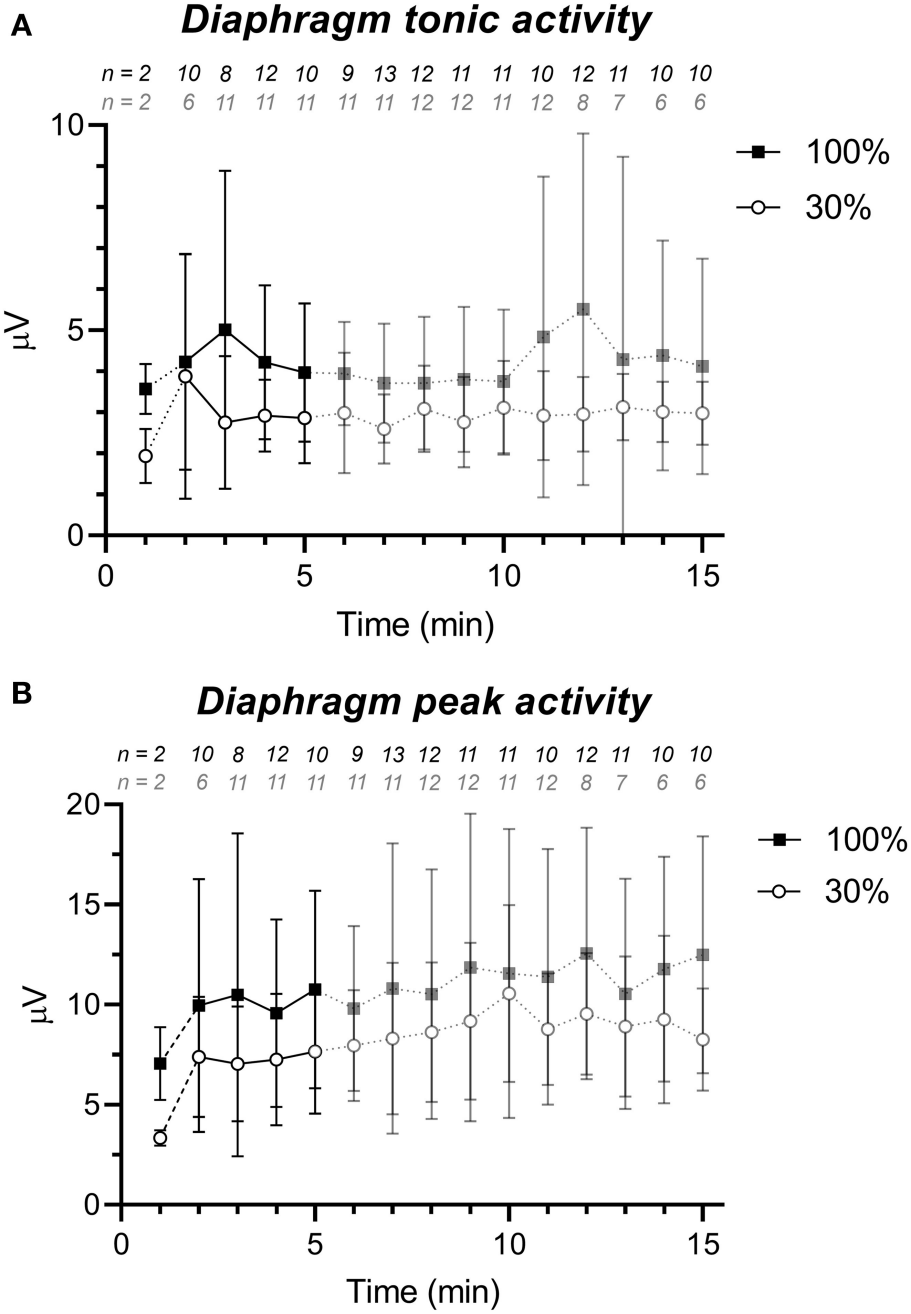
dEMG signal strength parameters (mean ± standard deviation) for the 30% (white circles) and 100% group (black squares) showing a higher level of diaphragm activity in the 100% group. **(A)** tonic diaphragm activity and **(B)** peak diaphragm activity. The first line piece is dashed, due to the low sample size directly after birth. Data after 5 min is semi-transparent as this data was not taken into account in the statistical analysis. The numbers above the graph represent the number of infants of which dEMG data was available (after artifact removal) for that particular minute and group.

As shown in Figure 1 peak and tonic activity were consistently higher in the 100% group than in the 30% oxygen group. The average value of dEMG_ton_ in the first 5 min after birth was significantly higher in the 100% group compared to the 30% group (4.3 ± 2.1 μV vs. 2.9 ± 1.0 μV; *p* = 0.047). The dEMG_peak_ value was also higher in the 100% group compared to the 30% group but this difference did not reach statistical significance (10.2 ± 5.1 μV vs. 7.2 ± 2.7 μV for the 100 and 30% oxygen group, respectively, *p* = 0.08). Average dEMG_amp_ (6.5 ± 4.1 μV vs. 4.2 ± 2.5 μV; *p* = 0.11) and dEMG_AUC_ (1.8 ± 1.0 μ*V* ·*s* vs. 1.3 ± 0.7 μ*V* ·*s*; *p* = 0.19) did not show statistically significant differences in the first 5 min after birth, between the high and the low oxygen group.

With respect to the dEMG derived breathing pattern, the two groups did not show differences in inspiratory and expiratory time. Overall respiratory rate was also similar in both groups (average mean RR in the first 5 min 34 ± 9 vs. 32 ± 10 breaths/min for the 100 and 30% oxygen group, respectively, *p* = 0.47).

## Discussion

In this study we demonstrated that a high level of oxygen administered to preterm infants directly after birth resulted in a consistently higher level of diaphragm activity compared to a low FiO_2_. No differences were found in inspiration time, expiration time, and respiratory rate.

We hypothesize that the higher diaphragmatic activity in the 100% group may, in part, explain the previously reported higher tidal volumes when administering 100% of oxygen compared to 30% of oxygen (9). First, both the higher peak and tonic diaphragmatic activity in the 100% oxygen group may assist in lung aeration after birth. A higher peak diaphragmatic activity could facilitate air entry in the fluid-filled lung during the first minutes of life. On top of that, an increased tonic diaphragmatic activity has been associated with an improved functional residual capacity (FRC) and thus retainment of inhaled air during pulmonary transition (15, 16). As shown by pre-clinical studies, more effective lung aeration can result in higher tidal volumes (17). Second, the higher diaphragmatic activity itself affects tidal volume. A recent study exploring the effect of caffeine on diaphragmatic activity and tidal volume showed a positive association between these two parameters (18).

Other non-diaphragm related factors may also contribute to a higher tidal volume when exposing infants to a higher FiO_2_. It has been shown that oxygen supports the development of a more stable breathing pattern and improves coordination of opening and closure of the glottis (19). These factors result in more efficient non-invasive respiratory support, which can impact both tidal volume and FRC.

The effect of oxygen on diaphragm activity was fast as the 100% oxygen group already had a higher level of diaphragm activity compared to the 30% group, in the first minutes of life. Furthermore, mean peak and tonic diaphragmatic activity remained higher on average in the 100% oxygen group at every time point up to 15 min after birth, even though dEMG_peak_ did not reach statistical significance.

The diaphragm activity in the 100% group remained higher until 15 min after birth, while the difference in the administered FiO_2_ and SpO_2_ between the groups decreased over time (9). This might indicate that the effect of the initial oxygen exposure persists after weaning of the FiO_2_ in the 100% group. This effect could be a direct result of the FiO_2_, or indirect (through improved overall oxygenation) as the 100% group became normoxic earlier in time (9). The administered FiO_2_ could be sensed by oxygen sensitive neuroendocrine cells present in the pulmonary epithelium, but current evidence on the role of these receptors during the transition from intra- to extra-uterine life is limited (20). It is also known that hypoxia inhibits both the neural output from the brainstem [through e.g., ATP and adenosine pathways (21)] and influences muscle performance itself (22). However, as FiO_2_ directly affects SpO_2_ and these parameters go hand in hand with each other it is impossible to determine whether the effect of the initial FiO_2_ on diaphragm activity is direct or indirect.

In general, the variability in diaphragm activity between infants was high. This finding might explain the lack of significance in dEMG_amp_ and dEMG_AUC_. There be may several reasons to explain this finding. First, the signal strength may be influenced by the varying electrode-diaphragm distance and skin-electrode interface between infants. The latter is also affected by differences in the wetness of skin between infants, which influences electrode adhesiveness and thereby signal strength (11). Second, similar to the *in-utero* situation, there is a physiological variation in breathing pattern in preterm infants shortly after birth (23). All these factors contribute to the variability in diaphragm activity between infants and thus make it difficult to obtain reference values for diaphragm activity. Larger studies are needed to determine these normative values. Meanwhile, dEMG can be used to monitor the trend in diaphragm activity over time and to determine the effect of changing the level of respiratory support in the DR.

Studies assessing diaphragmatic activity in the DR are limited. A recent study on the feasibility of dEMG to monitor heart rate and respiratory rate in the delivery room also assessed diaphragmatic activity (11). This study showed a trend to decreasing peak and tonic activity over time. The relatively stable diaphragm activity in the current study does not seem entirely consistent with this feasibility study. However, there were important differences between these studies which may explain this inconsistency. First, infants in the current study were less mature than those included in the feasibility study (mean gestational age 28 vs. 32 weeks). Less mature infants have a more compliant chest wall and therefore need to maintain a high level of muscle activity to open the lungs and keep them open (11, 24, 25). Second, the trend in diaphragmatic activity in the feasibility study was measured over a longer time (30 min) and larger intervals (5 min), which hampers direct comparison.

### Study Limitations

This study has several limitations that need to be addressed. First, there was an inevitable delay in placing the electrodes after birth, which was not part of the normal neonatal resuscitation program. As a result, diaphragmatic activity during the first minute of transition was missed in most of the included infants. However, as this was the case in both oxygen groups, it is unlikely to have biased the results. In addition, it should be mentioned that dEMG was only recorded in case research personnel and equipment were available, which could have introduced a selection bias. Third, although statistically not significant, the 30%-group received more IPPV than the 100% group, which may have affected the diaphragmatic activity (11). Finally, the unanticipated large variability in diaphragm activity and the relatively small sample size may have compromised the power of our study. Future studies should take this into account.

### Clinical Implication

The results of this study emphasize the importance of the diaphragm in pulmonary transition. Stimulating diaphragmatic activity with higher concentrations of inspired oxygen seems to facilitate lung aeration and improve tidal ventilation. The concomitant improvement in oxygenation might prove important as hypoxemia <80% at 5 min after birth is associated with a higher risk for mortality and intraventricular hemorrhage (26). Our study results show that diaphragm activity is influenced by the initial level of oxygen exposure. Whether a similar effect on diaphragm activity could be found with an intermediate level of oxygen (e.g., FiO_2_ 0.5–0.6) might be interesting to investigate, as it could reduce potential oxidative effects. Therefore, evaluation of diaphragm activity could contribute to the discussion on which initial level of oxygen to use in the first minutes of life.

## Conclusion

Electrical activity of the diaphragm, measured with transcutaneous electromyography, showed significantly higher tonic activity and a trend to higher peak activity in preterm infants resuscitated with 100% compared to 30% oxygen at birth. This finding suggests that the observed improvement in tidal breathing when using 100% instead of 30% oxygen is, at least partly, mediated by an oxygen dependent increase in diaphragmatic activity.

## Data Availability Statement

The datasets generated for this study are available on request to the corresponding author.

## Ethics Statement

The studies involving human participants were reviewed and approved by Commissie Medische Ethiek, cme registration: P17.209, cme@lumc.nl. Written informed consent to participate in this study was provided by the participants' legal guardian/next of kin. Written informed consent was obtained from the individual(s), and minor(s)' legal guardian/next of kin, for the publication of any potentially identifiable images or data included in this article.

## Author Contributions

RL, FJ, AK, JD, AP, and JH conceptualized the study. RL and AS analyzed the data and wrote the first version of the manuscript. RL submitted the paper. All authors contributed to the interpretation of the study results and critically reviewed and contributed to the final draft of the manuscript.

## Conflict of Interest

The authors declare that the research was conducted in the absence of any commercial or financial relationships that could be construed as a potential conflict of interest.
